# The C-Terminus Tail Regulates ERK3 Kinase Activity and Its Ability in Promoting Cancer Cell Migration and Invasion

**DOI:** 10.3390/ijms21114044

**Published:** 2020-06-05

**Authors:** Lobna Elkhadragy, Hadel Alsaran, Weiwen Long

**Affiliations:** 1 Department of Biochemistry and Molecular Biology, Boonshoft School of Medicine, Wright State University, Dayton, OH 45435, USA; lobna@uic.edu (L.E.); alsaran.2@wright.edu (H.A.); 2 Department of Radiology, University of Illinois at Chicago, Chicago, IL 60612, USA

**Keywords:** extracellular signal-regulated kinase (ERK), ERK3, mitogen-activated protein kinase (MAPK), MAPK6, septin 7, intramolecular regulation, cell migration, cell invasion

## Abstract

Extracellular signal-regulated kinase 3 (ERK3) is an atypical member of the mitogen-activated protein kinase (MAPK) family. It harbors a kinase domain in the N-terminus and a long C-terminus extension. The C-terminus extension comprises a conserved in ERK3 and ERK4 (C34) region and a unique C-terminus tail, which was shown to be required for the interaction of ERK3 with the cytoskeletal protein septin 7. Recent studies have elucidated the role of ERK3 signaling in promoting the motility and invasiveness of cancer cells. However, little is known about the intramolecular regulation of the enzymatic activity and cellular functions of ERK3. In this study, we investigated the role of the elongated C-terminus extension in regulating ERK3 kinase activity and its ability to promote cancer cell migration and invasion. Our study revealed that the deletion of the C-terminus tail greatly diminishes the ability of ERK3 to promote the migration and invasion of lung cancer cells. We identified two molecular mechanisms underlying this effect. Firstly, the deletion of the C-terminus tail decreases the kinase activity of ERK3 towards substrates, including the oncogenic protein steroid receptor co-activator 3 (SRC-3), an important downstream target for ERK3 signaling in cancer. Secondly, in line with the previous finding that the C-terminus tail mediates the interaction of ERK3 with septin 7, we found that the depletion of septin 7 abolished the ability of ERK3 to promote migration, indicating that septin 7 acts as a downstream effector for ERK3-induced cancer cell migration. Taken together, the findings of this study advance our understanding of the molecular regulation of ERK3 signaling by unraveling the role of the C-terminus tail in regulating ERK3 kinase activity and functions in cancer cells. These findings provide useful insights for the development of therapeutic agents targeting ERK3 signaling in cancer.

## 1. Introduction

Mitogen-activated protein kinases (MAPKs) are a family of serine/threonine kinases that mediate intracellular signaling and regulate various cellular processes. Based on their structures and activation pathways, they are classified into conventional and atypical MAPKs [1]. Conventional MAPKs include extracellular signal-regulated kinases 1/2 (ERK1/2), p38, and ERK5. Atypical MAPKs include ERK3, ERK4, and ERK7/8 [1]. ERK3 (MAPK6) has an elongated C-terminal extension that comprises about 400 amino acids. The first 150 residues of this extension are nearly 50% identical to a region in ERK4, and hence this region is called the conserved in ERK3 and ERK4 (C34) domain. Downstream of the C34, there is an unusually long tail of 240 amino acids that does not share significant homology with any known functional domains or motifs. Although the C-terminal tail is unique for ERK3, it is conserved throughout vertebrate evolution, suggesting that it has important functions [2]. Other MAPKs that have an elongated C-terminal extension beyond their kinase domains are ERK4, ERK5, and ERK7. While little is known about the intramolecular regulation of the kinase activity in ERK3 and ERK4, the C-terminal extensions of ERK5 and ERK7 appear to have opposite roles in the regulation of kinase activity. In ERK5, the C-terminal domain has an autoinhibitory role, as truncation of this region drastically increases the kinase activity of ERK5 [3]. On the contrary, the deletion of the C-terminal domain of ERK7 reduces its kinase activity [4].

ERK3 is ubiquitously expressed in human tissues and plays a role in several physiological processes [2]. For example, ERK3 increases neuronal dendritic spine formation via interaction with the cytoskeletal protein septin 7 [5]. Moreover, ERK3 is required for T-cell activation and proper thymic positive selection [6,7], and it promotes the migration and proliferation of endothelial cells [8]. ERK3 also has important roles in cancers. Both in vitro and in vivo studies have demonstrated the ability of ERK3 to increase the migration and invasion of lung cancer cells [9]. A downstream mediator for the invasiveness-promoting role of ERK3 in lung cancer cells is steroid receptor coactivator-3 (SRC-3) [9]. SRC-3 is a transcriptional coactivator that has oncogenic activity and is overexpressed in multiple human cancers [10]. In line with its role in lung cancer cells, ERK3 promotes the migration of breast cancer cells and head and neck cancer cells [11,12]. Recent studies have shed light on some molecular features that regulate the role of ERK3 in cancer. For example, the kinase activity and Ser^189^ phosphorylation are both crucial for the invasiveness-promoting role of ERK3 in cancer cells [13]. Ser^189^ is a single phospho-acceptor site in the activation motif of ERK3. It can be phosphorylated by upstream kinases such as group I p21-activated protein kinases (PAKs) or as a result of ERK3 autophosphorylation [14,15,16]. Interestingly, the mutation of the catalytic site decreases, but does not abolish, the ability of ERK3 to promote cancer cell invasiveness, demonstrating the existence of both kinase-dependent and kinase-independent signaling mechanisms for ERK3 in cancer cells [13]. 

Currently, little is known about the importance of the C-terminus extension in kinase activity or functions of ERK3. The objective of the current study is to advance our understanding of the intramolecular regulation of ERK3 by its unique elongated C-terminus extension. To dissect its role in regulating the enzymatic activity and function of ERK3 in cancer cells, we investigated the effect of truncating the C-terminal tail or the entire C-terminal extension on the kinase activity of ERK3 towards known substrates, as well as on its ability to promote cancer cell migration and invasion. 

## 2. Results

### 2.1. The C-Terminal Tail Is Important for the Migration- and Invasion-Promoting Abilities of ERK3 in Lung Cancer Cells

**Figure 1:**
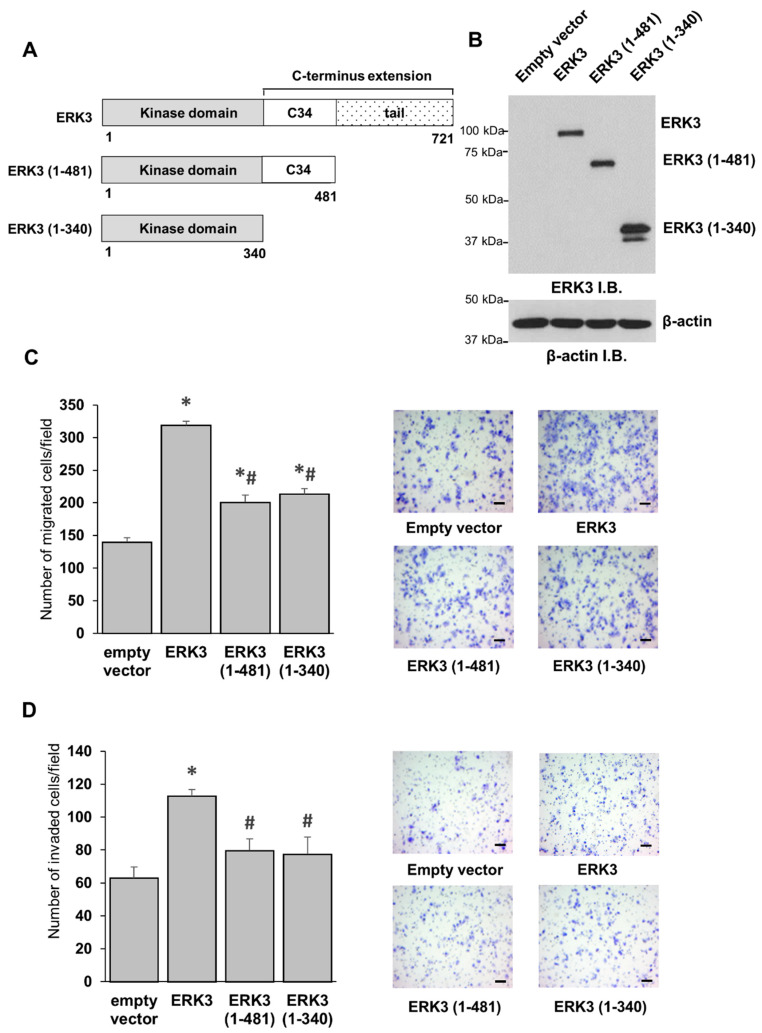
The C-terminus tail is important for the migration- and invasion-promoting abilities of extracellular signal-regulated kinase 3 (ERK3) in lung cancer cells. (**A**) Schematic representation of full length and deletion mutant ERK3 proteins. The conserved in ERK3 and ERK4 (C34) domain. (**B**) H1299 cells were transfected with equimolar concentrations of an empty vector, ERK3, ERK3 (1-481) or ERK3 (1-340) plasmids. Two days post-transfection, the cells were lysed and analyzed by Western blotting using an anti-ERK3 monoclonal antibody. Immunoblots (I.B.). (**C**) The migration ability of H1299 cells with the overexpression of the indicated plasmids was determined by transwell migration assay. Values in the bar graphs represent mean ± standard error (SE) (*n* ≥ 6 fields). *: significantly different compared to empty vector (*p* < 0.001); #: significantly different compared to ERK3 (*p* < 0.0001) by one-way ANOVA. Representative images of migrated cells stained with crystal violet are shown. Scale bar, 100 μm. (**D**) Transwell Matrigel invasion assay of H1299 cells with the overexpression of each plasmid as indicated. Values in the bar graphs represent mean ± SE (*n* ≥ 6 fields). *: significantly different compared to empty vector (*p* < 0.001); #: significantly different compared to ERK3 (*p* < 0.05) by one-way ANOVA. Representative images of invaded cells stained with crystal violet are shown. Scale bar, 100 μm.

One of the key structural properties that sets ERK3 apart from most of the conventional MAPKs is the presence of a long C-terminal extension. It remains unclear if this extension regulates the activity or functions of ERK3. The C-terminal extension of ERK3 comprises the C34 domain, which spans amino acids 341 to 481, and a tail comprising 240 amino acids, from amino acid 482 to 721 (Figure 1A). To determine if the C-terminus extension was involved in ERK3-induced cancer cell invasiveness, we overexpressed full length ERK3 or ERK3 deletion mutants in H1299 lung cancer cells, and validated the protein expression by Western blotting (Figure 1B). The deletion mutant ERK3 (1-481) comprised the kinase domain and the C34 domain, but lacked the C-terminal tail. The other deletion mutant, ERK3 (1-340), comprised only the kinase domain. In agreement with previous studies [9,13,17], the overexpression of ERK3 increased the migration and invasion of lung cancer cells (Figure 1C,D). Both the deletion mutants, ERK3 (1-481) and ERK3 (1-340), had a significantly reduced ability to promote migration and invasion compared to full length ERK3 (Figure 1C,D). Notably, there was no significant difference between ERK3 (1-340) and ERK3 (1-481), suggesting that the C-terminal tail (amino acids 482-721), but not the C34 domain, is critical for the ability of ERK3 to promote cancer cell migration and invasion. 

### 2.2. The C-Terminus Extension Is Important for In Vitro Kinase Activity of ERK3 

The kinase activity was shown to be—at least partly—important for the ability of ERK3 to promote migration and invasion in lung cancer cells [13]. Hence, we sought to investigate if the functional role of the C-terminal tail of ERK3 in cancer cells was linked to the regulation of enzymatic activity. To test the effect of C-terminal truncations on the enzymatic activity of ERK3, we purified ERK3 deletion mutant proteins and compared their kinase activity with that of full length ERK3 towards known substrates by a radioactive in vitro kinase assay. 

**Figure 2:**
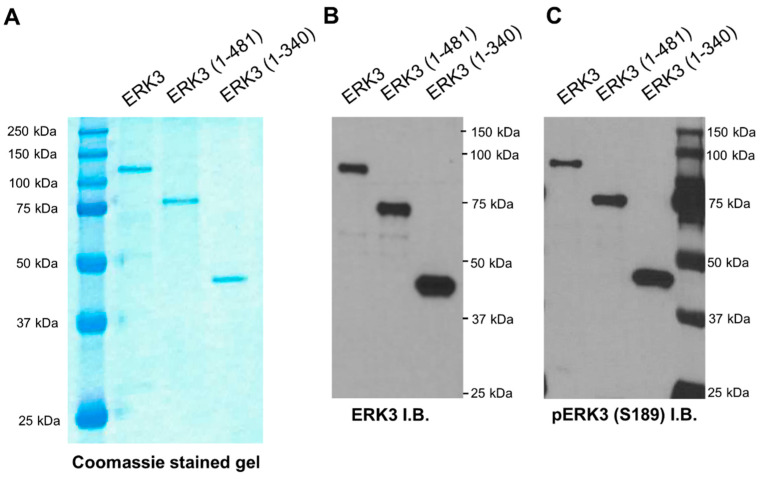
Purification of full length or deletion mutant ERK3 proteins by expression and immunoprecipitation from 293T cells. (**A**) 293T cells were transfected with plasmids expressing HA-ERK3, HA-ERK3 (1-481) or HA-ERK3 (1-340). Two days post-transfection, exogenously expressed ERK3 proteins were immunoprecipitated using HA antibody-conjugated agarose beads and eluted with HA peptide. For each purified protein, 250 ng was analyzed by SDS-PAGE gel followed by Coomassie blue staining. (**B**,**C**) Western blot analysis of proteins purified from mammalian cells using an anti-ERK3 antibody (**B**) or an anti-phsopho-ERK3 (S189) antibody (**C**). Immunoblots (I.B.).

**Figure 3:**
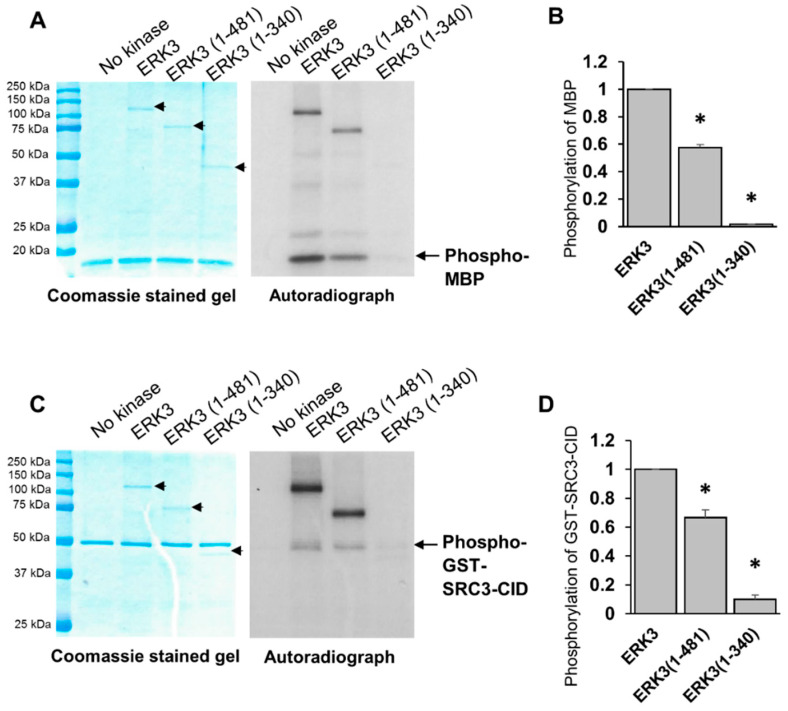
Truncation of the C-terminus extension decreases the in vitro kinase activity of ERK3 towards myelin basic protein (MBP) and steroid receptor co-activator 3 (SRC-3) substrates. (**A**) In vitro kinase assay for full length or deletion mutant ERK3 proteins purified from mammalian 293T cells using MBP as a substrate. The assay was performed by incubating 40 nM of each ERK3 protein with 1 μg of recombinant MBP in kinase assay buffer containing (γ-^32^P)-ATP. The reaction products were analyzed by SDS-PAGE and autoradiography. Total level of MBP substrate in each reaction is shown by Coomassie staining (left panel). ERK3 proteins in the Coomassie stained gel are marked with arrowheads. Please note that ERK3 proteins are barely seen in the Coomassie-stained gels due to their small amounts. The substrate phosphorylation was detected by autoradiography and marked with arrow (right panel). (**B**) Quantification of MBP phosphorylation by full length or deletion mutant ERK3 proteins. For the purpose of comparison, the normalized phosphorylation level of MBP by ERK3 was arbitrarily set as 1.0. The bar graph represents the mean ± SE of three independent experiments. * *p* < 0.001 by one-way ANOVA. (**C**) In vitro kinase assay for full length or deletion mutant ERK3 proteins purified from mammalian 293T cells using glutathione S-transferase (GST)-SRC-3-CBP-interacting domain (CID) as a substrate. The assay was performed by incubating 40 nM of each ERK3 protein with 1 μg of recombinant GST-SRC-3-CID in kinase assay buffer containing (γ-^32^P)-ATP. Total level of substrate in each reaction is shown by Coomassie staining (left panel) and substrate phosphorylation is detected by autoradiography and marked with arrow (right panel). ERK3 proteins in the Coomassie stained gel are marked with arrowheads. (**D**) Quantification of GST-SRC-3-CID phosphorylation by full length or deletion mutant ERK3 proteins. The normalized phosphorylation level of GST-SRC-3-CID by ERK3 was arbitrarily set as 1.0. The bar graph represents the mean ± SE of three independent experiments. * *p* < 0.005 by one-way ANOVA.

HA-tagged ERK3 deletion mutant proteins were expressed and immunoprecipitated from mammalian 293T cells. The isolated proteins were analyzed by Coomassie blue staining of SDS-PAGE gel and Western blotting analysis. The full length ERK3, ERK3 (1-481) and ERK3 (1-340) migrated at around 100 kDa, 75 kDa, and 42 kDa, respectively (Figure 2A,B). Immunoblotting with an antibody specific to ERK3 phosphorylated on Ser^189^ demonstrated a similar level of phosphorylation of the three proteins at Ser^189^ in the activation loop (Figure 2C). Next, we compared the kinase activities of these purified proteins towards ERK3 substrates. Myelin basic protein (MBP) is a non-specific MAPK substrate that is phosphorylated by ERK3 in vitro [18]. Steroid receptor co-activator-3 (SRC-3) is a substrate for ERK3 that mediates its invasiveness-promoting role in lung cancer [9]. As ERK3 phosphorylates SRC-3 on Ser^857^ residue which locates within the CREB-binding protein (CBP)-interacting domain (CID), we purified glutathione S-transferase (GST)-tagged SRC-3-CID fragment and used it as a substrate [9]. The truncation of the C-terminal tail decreased the kinase activity of ERK3 towards MBP or SRC-3 substrates by about 40% (compare ERK3 (1-481) to full length ERK3, Figure 3). Interestingly, truncation of the entire C-terminus extension (both C34 domain and C-terminal tail) resulted in a drastic decrease in ERK3 activity towards each of the substrates (compare ERK3 (1-340) to full length ERK3, Figure 3). Consistent with their effects on substrate phosphorylation, both of the ERK3 deletion mutant proteins exhibited reduced autophosphorylation in the in vitro kinase assays. Taken together, these results show that the entire C-terminal extension, comprising the C34 domain and tail, is important for the kinase activity of ERK3.

### 2.3. The C-terminus Tail Is Not Involved in the Kinase-Independent Signaling of ERK3 in Cancer Cells 

**Figure 4:**
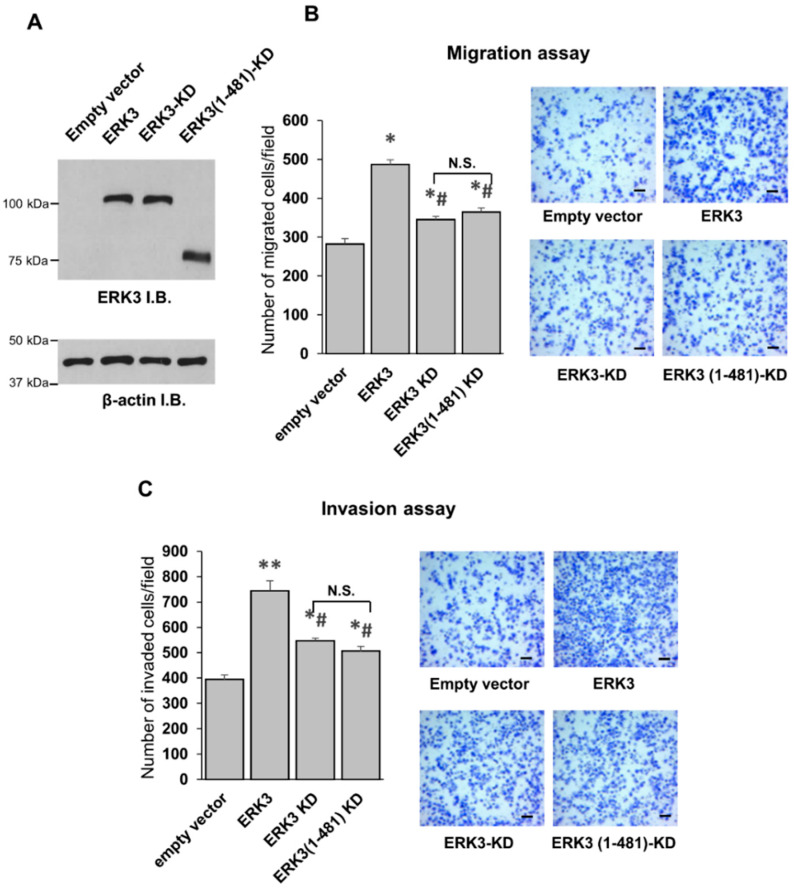
The C-terminus tail is important for the invasiveness-promoting ability of ERK3 through a kinase-dependent mechanism. (**A**) H1299 cells were transfected with an empty vector, ERK3, ERK3 kinase-dead (ERK3-KD) or ERK3 (1-481)-KD plasmids. Two days post-transfection, the cells were lysed and analyzed by Western blotting using an anti-ERK3 monoclonal antibody. Immunoblots (I.B.). (**B**) The migration ability of H1299 cells with overexpression of the indicated plasmids was determined by transwell migration assay. Values in the bar graphs represent mean ± SE (*n* ≥ 6 fields). *: significantly different compared to empty vector (*p* < 0.005); #: significantly different compared to ERK3 (*p* < 0.0001); not significant (N.S.) by one-way ANOVA. Representative images of migrated cells stained with crystal violet are shown. Scale bar, 100 μm. (**C**) The invasion ability of H1299 cells with overexpression of the indicated plasmids was determined by transwell Matrigel invasion assay. Values in the bar graphs represent mean ± SE (*n* ≥ 6 fields). **: significantly different compared to empty vector (*p* < 0.0001); *: significantly different compared to empty vector (*p* < 0.05); #: significantly different compared to ERK3 (*p* < 0.0001) by one-way ANOVA. Representative images of invaded cells stained with crystal violet are shown. Scale bar, 100 μm.

A kinase-independent role for ERK3 in promoting cancer cell migration and invasion has been recently revealed [11,13]. However, little is known about the residue(s) or region(s) in ERK3 responsible for this effect. To investigate if the C-terminus tail was involved in the kinase-independent signaling of ERK3, we tested the effect of the truncation of the C-terminal tail on the migration- and invasion-promoting abilities of a catalytically inactive ERK3 mutant (Figure 4). If the C-terminus tail was important for the kinase-independent effects, truncation of this region would diminish ERK3 kinase dead (ERK3-KD)-induced cancer cell invasiveness. 

Consistent with previous findings, while the catalytically inactive ERK3 mutant protein (ERK3-KD) has lower migration- and invasion-promoting abilities than ERK3 (compare ERK3-KD to ERK3, Figure 4), cells overexpressing ERK3-KD showed significantly higher migration and invasion compared to the control cells expressing an empty vector (compare ERK3-KD to empty vector, Figure 4). The truncation of the C-terminal tail did not alter the effect of ERK3-KD on cancer cell migration and invasion (compare ERK3 (1-481)-KD to ERK3-KD, Figure 4), suggesting that the C-terminal tail is not important for the kinase-independent role of ERK3 in cancer cells. These results show that the role of the C-terminal tail in promoting cancer cell invasiveness is linked with its effect on the kinase activity of ERK3. 

### 2.4. Septin 7 Is a Downstream Effector for ERK3-Induced Cancer Cell Migration

**Figure 5:**
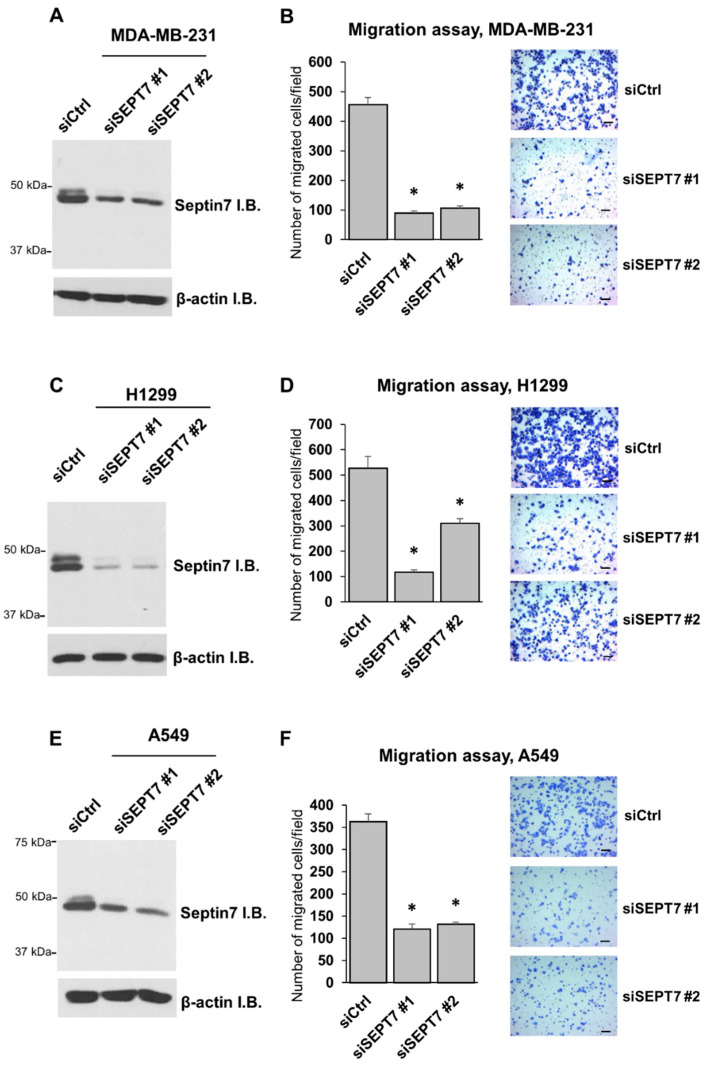
Knockdown of septin 7 decreases cancer cell migration. (**A**) MDA-MB-231 breast cancer cells were transfected with 30 nM non-targeting siRNA (siCtrl) or siRNAs targeting septin 7: siSEPT7 #1 or siSEPT7 #2. Two days post-transfection, the cells were harvested and the knockdown was confirmed by Western blotting using an anti-Septin 7 antibody. (**B**) The migration ability of MDA-MB-231 with knockdown of septin 7 was determined by transwell migration assay. Values in the bar graphs represent mean ± SE (*n* = 6 fields). *: significantly different compared to siCtrl (*p* < 0.0001) by one-way ANOVA. Representative images of migrated cells stained with crystal violet are shown. Scale bar, 100 μm. (**C**) Western blotting analysis of septin 7 protein level in H1299 lung cancer cells transfected with 30 nM of the indicated siRNA for two days. (**D**) Transwell migration assay for H1299 cells with knockdown of septin 7. Values in the bar graphs represent mean ± SE (*n* = 6 fields). *: significantly different compared to siCtrl (*p* < 0.001) by one-way ANOVA. Scale bar, 100 μm. (**E**) Western blotting analysis of septin 7 protein level in A549 lung cancer cells transfected with 30 nM of the indicated siRNA for two days. (**F**) Transwell migration assay for A549 cells with knockdown of septin 7. Values in the bar graphs represent mean ± SE (*n* = 6 fields). *: significantly different compared to siCtrl (*p* < 0.0001) by one-way ANOVA. Scale bar, 100 μm.

Septin 7 is a GTP-binding protein that is involved in structural re-modelling processes of cells [19]. Previous studies showed that the interaction of septin 7 with the C-terminus extension of ERK3 results in the formation of a ternary complex that regulates neuronal cytoskeleton and increases neuronal dendritic spine formation in primary hippocampal neurons [5]. As septin 7 interacts with the C-terminal tail of ERK3, and we have demonstrated the importance of this region for invasiveness-promoting ability of ERK3, we sought to investigate the possible involvement of septin 7 in the role of ERK3 in cancer cells. Firstly, we tested the effect of knockdown of septin 7 on the migration of breast cancer cells and lung cancer cells. Knockdown of septin 7 by two different siRNAs was confirmed in three cell lines by Western blotting (Figure 5A,C,E). Knockdown of septin 7 decreased cancer cell migration of MDA-MB-231 breast cancer cells, H1299, and A549 lung cancer cells (Figure 5B,D,F).

**Figure 6:**
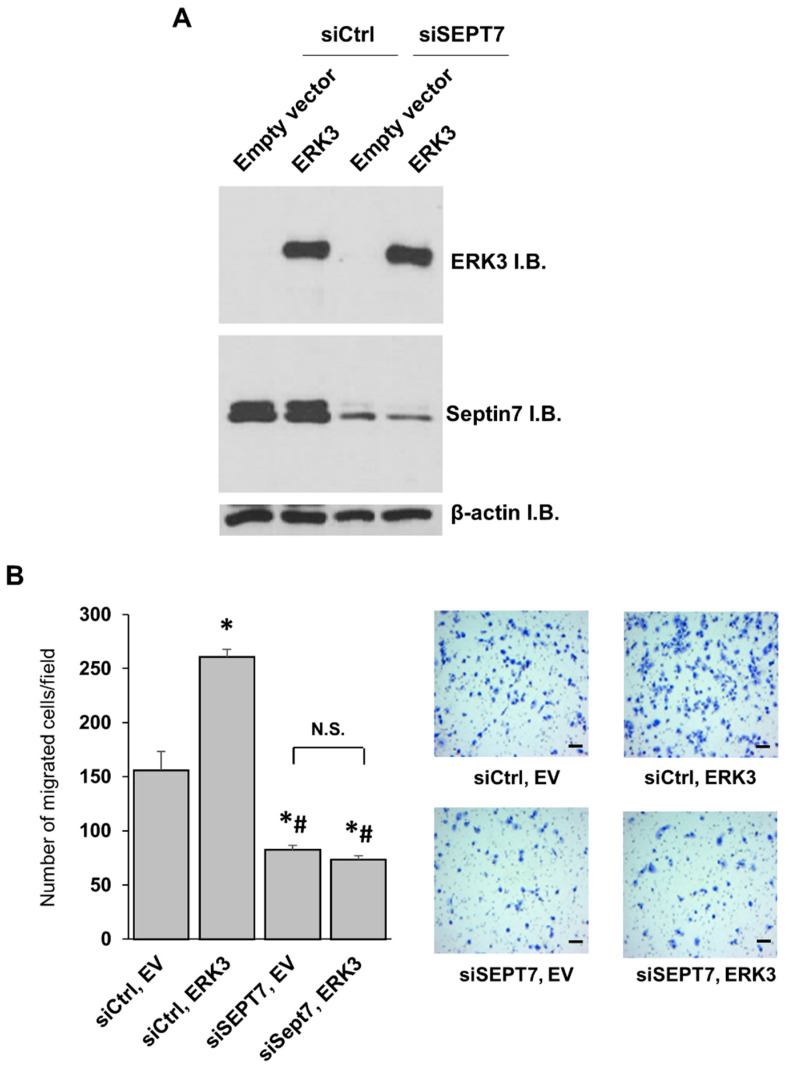
Septin 7 is a downstream effector for ERK3-induced cancer cell migration. (**A**) H1299 cells were co-transfected with an empty vector or ERK3 plasmid, and siCtrl or siRNA targeting Septin 7 (siSEPT7 #1). Two days post-transfection, the cells were lysed and analyzed by Western blotting using anti-ERK3 and anti-Septin 7 antibodies. (**B**) The migration ability of H1299 cells with transfection of each indicated plasmid and siRNA was determined by transwell migration assay. Values in the bar graphs represent mean ± SE (*n* ≥ 6 fields). *: significantly different compared to siCtrl, empty vector (*p* < 0.0001); #: significantly different compared to siCtrl, ERK3 (*p* < 0.0001); not significant (N.S.) by one-way ANOVA. Representative images of migrated cells stained with crystal violet are shown. Scale bar, 100 μm. Empty vector (EV).

Next, we wanted to test if septin 7 was a downstream mediator for ERK3 function in promoting cancer cell motility. As expected, overexpression of ERK3 increased migration (compare siCtrl/ERK3 to siCtrl/empty vector, Figure 6A,B) and knockdown of septin 7 decreased migration of lung cancer cells (compare siSEPT7/empty vector to siCtrl/empty vector, Figure 6A,B). Importantly, the knockdown of septin 7 abolished the ability of ERK3 to promote lung cancer cell migration (compare siSEPT7/ERK3 to siSEPT7/empty vector, Figure 6A,B). These results identify septin 7 as a downstream effector for the migration-promoting role of ERK3 in cancer cells. 

## 3. Discussion

A characteristic structural feature of ERK3 is the presence of a relatively long C-terminus extension beyond its N-terminal kinase domain. The first region of this C-terminus extension is highly conserved in ERK3 and ERK4, hence it is called the C34 domain, whereas the remaining C-terminal tail is unique to ERK3 and does not share similarity with known domains in other proteins. It is currently unclear what roles this C-terminus extension plays in the regulation of the kinase activity and the cellular functions of ERK3. To address these questions, we investigated the effects of truncating regions in the C-terminus extension on the ability of ERK3 to promote cancer cell invasiveness, and on the in vitro kinase activity of ERK3. Our study revealed that the C-terminus extension is crucial for regulating the enzymatic activity of ERK3. Truncation of the C-terminal tail decreased the kinase activity of ERK3 towards MBP and SRC-3 by about 40%, and truncation of the entire C-terminal extension decreased the kinase activity by more than 90%. Consistent with reducing the kinase activity, C-terminal truncations also reduced ERK3 autophosphorylation in the in vitro kinase assay. Notably, C-terminal truncation did not affect cellular ERK3 Ser^189^ phosphorylation as determined by Western blotting of cell lysates. The phosphorylation of ERK3 Ser^189^ in cultured cells can result from autophosphorylation and/or transphosphorylation by upstream kinase(s), whereas ERK3 phosphorylation detected in the in vitro kinase assay mainly results from autophosphorylation due to the absence of other kinases. These results suggest that the C-terminus extension is important for the kinase activity of ERK3, but does not affect transphosphorylation of ERK3 by upstream kinases in cells, and that Ser^189^ phosphorylation in cells is mainly owing to transphosphorylation. 

There is an important question that is yet to be answered: how does the C-terminus extension regulate the kinase activity of ERK3? Structural studies would be instrumental in answering this question. Attempts have been made to elucidate the structure of the bacterially expressed kinase domain of ERK3 by X-Ray crystallography (Protein Data Bank (PDB), ID: 2I6L, unpublished). However, the crystal structure of the kinase domain alone does not tell us the involvement of the C-terminus extension in the conformation of the full length ERK3 protein. We speculate three potential mechanisms for regulation of ERK3 kinase activity by its C-terminus extension. The first mechanism involves an intramolecular regulation of the kinase domain by the C-terminus through direct interactions, which facilitates the formation of an activated structural conformation of ERK3. Secondly, the C-terminus could be required for recruiting proteins and/or other molecules which activate ERK3. Thirdly, the C-terminus may facilitate substrate binding. These three mechanisms may exist simultaneously. As to the first mechanism, a hint for intramolecular interactions within ERK3 comes from studies of its ubiquitination [20,21]. These studies demonstrated that even though ERK3 is ubiquitinated on its free N-terminus, the addition of large tags on its C-terminus can protect it from ubiquitination-mediated degradation, suggesting the existence of interactions between the N- and C-termini. In addition, previous work has suggested that ERK3 proteins form homodimers, yet it is unclear how the homodimers are configured [22]. Further work will be necessary to elucidate the underlying molecular mechanism by which the C-terminus extension regulates the kinase activity of ERK3. 

Our study has also uncovered an important function for the C-terminus tail of ERK3 in its invasiveness-promoting ability in cancer cells. An ERK3 deletion mutant lacking the C-terminus tail had a significantly decreased ability to promote the migration and invasion of lung cancer cells. Given that this deletion mutant protein has reduced kinase activity, these findings are in line with the importance of ERK3 kinase activity for promoting cancer cell migration and invasion. ERK3 also promotes cell motility via a kinase-independent mechanism [11,13]. We found that the deletion of the C-terminus tail did not affect the ability of the kinase-dead form of ERK3 (ERK3-KD) to promote cancer cell migration, suggesting that the C-terminus tail might not be involved in the kinase-independent role of ERK3, but rather that it contributes to the ERK3-induced cell motility in a kinase-dependent manner. 

Noteworthily, while the kinase domain alone (ERK3 (1-340)) has much less kinase activity in vitro than that of the fragment containing both the kinase domain and the C34 domain (ERK3 (1-481)), these two deletion mutant proteins showed similar effects on cancer cell migration and invasion. These results suggest that the C-terminal tail of ERK3 (amino acids 482-721), but not the C34 domain, is the important region in the C-terminus extension for the migration/invasion-promoting functions of ERK3. This could be attributed to a role of the C-terminus tail of ERK3 in recruiting substrates particularly involved in cancer cell motility and invasiveness. Indeed, our study revealed a role for the C-terminus tail in mediating the formation of ERK3/septin 7 complex and promoting cancer cell motility. Previous work has demonstrated the interaction between septin 7 and the C-terminus extension of ERK3 and the cooperative role of ERK3 and septin 7 in neuronal morphogenesis [5]. The role of septin 7 in cancers varies depending upon the cancer type: it increases the migration and invasion of breast cancer cells [23], but inhibits the proliferation, migration, and invasion of glioma cells [24,25,26]. Our study demonstrates the importance of septin 7 for the migration of lung cancer cells, and confirms previous findings about its role in breast cancer cells [23]. Importantly, we have found that septin 7 is a downstream mediator of the migration-promoting ability of ERK3 in lung cancer cells. Future work would be necessary to investigate the role of the ERK3/septin 7 complex in other cellular processes, and to reveal the molecular signaling mechanisms downstream of this complex. 

To conclude, in this study, we explored the previously unknown effects of the C-terminus extension on the kinase activity and functions of ERK3 in cancer cells. Our work uncovers the important role of the C-terminus extension (amino acids 341-721) in controlling the kinase activity of ERK3, and the important role of the C-terminus tail (amino acids 482-721) for the ability of ERK3 to promote cancer cell migration and invasion. We also demonstrate that knockdown of the cytoskeletal protein septin 7 decreases the motility of lung cancer cells, and identify septin 7 as a downstream effector for ERK3-induced cancer cell migration. Overall, our study reveals the intramolecular regulation of ERK3 activity and functions in cancer cells by the C-terminus extension. These findings are important for developing anti-cancer therapeutic strategies through the inhibition of ERK3 signaling.

## 4. Materials and Methods

### 4.1. Cell Culture

H1299 and A549 lung cancer cells were maintained in RPMI-1640 growth medium (Gibco/ThermoFisher Scientific, Waltham, MA, USA, catalog # A1049101). MDA-MB-231 breast cancer cells and 293T human embryonic kidney cells were maintained in DMEM (Gibco, catalog # 11965092). The growth media was supplemented with 10% fetal bovine serum (FBS, Gibco, catalog # 16000044) and 1% penicillin–streptomycin (Gibco, catalog # 15140122). All the cell lines were purchased from the American Type Culture Collection (ATCC), Manassas, VA, USA. 

### 4.2. Expression Plasmids

The mammalian expression constructs of ERK3 and ERK3-kinase dead (KD) with HA tags at the N-terminus (pSG5-HA-ERK3) were described previously [17]. ERK3-KD has mutations of K49/50A in the ATP binding site of ERK3. The expression constructs of ERK3 deletion mutants pSG5-HA-ERK3 (1-481) and pSG5-HA-ERK3 (1-340) were generated by PCR amplification of cDNA encoding ERK3 amino acids (1-481) or (1-340), respectively, using pSG5-HA-ERK3 as a template and primers with EcoRI sites (sequences listed in Table 1). Each PCR product was then inserted into pCR^®^ 2.1-TOPO^®^ TA vector (Invitrogen, ThermoFisher Scientific, Waltham, MA, USA, catalog # K450002) and digested with EcoRI (New England BioLabs, Ipswich, MA, USA, catalog # R0101S). The released fragments were subcloned into the EcoRI site of pSG5-HA-ERK3. pSG5-HA-ERK3 (1-481)-KD was generated similarly, but by using pSG5-HA-ERK3-KD as a template. The sequence of all the plasmids generated was verified by Sanger sequencing. The pGEX-4T-1-SRC-3-CID plasmid previously described was used for the bacterial expression of GST-SRC-3-CID, which contains amino acids 841–1080 of SRC-3 with a glutathione S-transferase (GST) tag [9]. 

### 4.3. Plasmid and siRNA Transfections

Transient transfections with plasmids were performed using Lipofectamine 3000 Reagent (Invitrogen, catalog # L3000008) or Fugene HD reagent (Active motif, Carlsbad, CA, USA, catalog # 32043), following the manufacturer’s instructions. Transient siRNA transfections were done using DharmaFECT Transfection Reagent (Dharmacon, Lafayette, CO, USA, catalog # T-2001-02), following the manufacturer’s instructions. The following siRNAs were purchased from ThermoFisher Scientific: Silencer select siRNAs targeting human septin 7: siSEPT7#1 (catalog # 4392420, assay ID s2741) and siSEPT7#2 (catalog # 4392420, assay ID s2743), and the Silencer Negative Control #1 (catalog # 4390843).

### 4.4. Western Blotting

Cells were lysed with EBC lysis buffer (50 mM Tris (pH 7.5), 150 mM NaCl, 0.5% NP-40, 1 mM Complete protease inhibitors (Roche Diagnostics, Basel, Switzerland, catalog # 11697498001) and 1 mM phosphatase inhibitor cocktail III (Sigma-Aldrich, St. Louis, MO, USA, catalog # P0044)). SDS-PAGE was done followed by transferring the proteins onto nitrocellulose membranes, and blocking the membranes with 5% non-fat milk in phosphate-buffered saline with tween 20 (PBS-T) for 30 min. Incubation with the primary antibodies was done overnight at 4 °C followed by 1-h incubation with the appropriate secondary antibodies at room temperature. The following primary antibodies were used: anti-ERK3 (Abcam, Cambridge, United Kingdom, catalog # ab53277), anti-phospho-ERK3 (S189), generated by our lab as described previously [9], anti-septin 7 (Immuno-Biological Laboratories, Minneapolis, MN, USA, catalog # 18991), and anti-β-actin (Sigma-Aldrich, catalog # A5316), and the following secondary antibodies were used: anti-mouse (Biorad, Hercules, CA, USA, catalog # 170-6516) and anti-rabbit (Biorad, catalog # 170-6515). The Western blots were visualized by chemiluminescence (ThermoFisher, catalog # 32109). β-actin was used as a loading control. 

### 4.5. Two-chamber Transwell Cell Migration and Invasion Assays 

Cell migration was analyzed using a modified two chamber transwell system (Corning, Corning, NY, USA, catalog # 353097), following the manufacturer’s instructions. Cells were detached by trypsin/EDTA, washed once with serum-free medium, and then resuspended in serum-free medium. Complete culture medium with 10% FBS was added to each bottom well. In total, 40,000 cells were added in each transwell insert and allowed to migrate for 16 h in a 37 °C cell incubator, then the cells in the upper surface of the transwell were removed using cotton swabs. The migrated cells attached on the undersurface were fixed with 4% paraformaldehyde for 15 min and stained with crystal violet solution (0.5% in water) for 10 min. Migrated cells were then photographed and quantified using ImageJ software. The quantitated migration ability was presented as the number of migrated cells per field. The cell invasion assay was performed by following the same procedures as those for the cell migration assay, except that 55,000 cells were placed in transwell inserts that were pre-coated with 1 mg/mL Growth Factor-Reduced Matrigel (Corning, catalog # 354230), and cells were allowed to invade for 16 h. 

### 4.6. Immunoprecipitation of ERK3 Protein from Mammalian Cells

Full length or deletion mutant HA-tagged ERK3 proteins were purified from mammalian cells as described previously [9]. The appropriate pSG5-HA-ERK3 plasmids were transfected in 293T cells. Two days post-transfection, cells were lysed and the protein lysate supernatants were incubated with anti-HA affinity agarose beads (Sigma-Aldrich, catalog # E6779) for 3 h. After washing the beads, the proteins were eluted using 120 ng/µL HA peptide (Sigma-Aldrich, catalog # I2149), then concentrated using an Amicon Ultra-0.5 Centrifugal Filter with a 10-kDa cutoff filter (Millipore-Sigma, Burlington, MA, USA, catalog # UFC501008). The protein purity was assessed by SDS-PAGE followed by staining with Coomassie blue solution (Expedeon, Heidelberg, Germany, catalog # ISB1L).

### 4.7. Recombinant Protein Expression

Recombinant GST-SRC-3-CID protein was purified as previously described [13]. *E. coli* BL-21 (DE3) cells were transformed with pGEX-4T-1-SRC-3-CID plasmid. A single colony of transformed cells was inoculated in LB medium and cultured at 37 °C. Induction of protein synthesis was done using 0.4 mM IPTG for 6 h at room temperature, then the cells were harvested by centrifugation at 5000× *g* for 20 min at 4 °C and stored at −20 °C until lysis. The cell pellet was lysed in Buffer A: 20 mM HEPES pH 7.6, 150 mM KCL, 10% glycerol, 1 mM Dithiothreitol (DTT), 0.1 mM phenylmethane sulfonyl fluoride (PMSF) and Complete Protease Inhibitor Cocktail (Roche) and sonicated. The clarified lysate was then incubated with Glutathione Sepharose 4B (GE Healthcare, Chicago, IL, USA, catalog # 17-0756-01) for 1.5 h at 4 °C and the proteins were eluted off the beads with 10 mM glutathione (Sigma, catalog #G4251) in 50 mM Tris HCL, pH 8.0. The concentration of the purified recombinant protein was determined by Bradford protein assay, and protein purity was assessed by SDS-PAGE followed by staining with Coomassie blue solution. The recombinant myelin basic protein (MBP) was a kind gift from Dr. Yong-jie Xu, Wright State University, Dayton, OH, USA.

### 4.8. In Vitro Kinase Assay

Radioactive in vitro kinase assays for ERK3 proteins were done as previously described [18]. Briefly, each in vitro kinase assay reaction contained 40 nM ERK3 protein, 1 µg substrate, 5 μCi (γ-^32^P)-ATP (Perkin Elmer, Waltham, MA, USA, catalog # NEG002Z) and 25 μM cold ATP (ThermoFisher, catalog # PV3227). The reactions were carried out at 30 °C for 30 min and then stopped by SDS sample buffer and boiling. Proteins were resolved by SDS-PAGE, stained with Coomassie blue solution and visualized by autoradiography. The quantification of substrate phosphorylation was determined by calculating the ratio of the band intensity of phosphorylated substrate in the autoradiograph over that of the corresponding total substrate protein in the Coomassie-stained gel.

### 4.9. Statistics

Data are expressed as mean ± standard error (SE). All experiments were repeated at least three times and a representative figure is presented. Statistical significance was determined by one-way analysis of variance (ANOVA) and a *p*-value of less than 0.05 was considered statistically significant.

## Figures and Tables

**Table 1:** Primers used in the study.

Primer	Sequence (5′ → 3′)
ERK3 (1-340) Forward	GGAATTCGGCAGAGAAATTTGAAAG
ERK3 (1-340) Reverse	GGAATTCTTAATCATCAACTTCATCTTCAATATG
ERK3 (1-481) Forward	GGAATTCGGCAGAGAAATTTGAAAG
ERK3 (1-481) Reverse	GGAATTCTTATTCTTTCCAATTGGAAAGATCTATA

## Abbreviations

ERK3	Extracellular signal-regulated kinase 3
MAPK	Mitogen-activated protein kinase
C34	Conserved in ERK3 and ERK4
SRC-3	steroid receptor co-activator 3
PAK	p21-activated protein kinase
MBP	Myelin basic protein
CBP	CREB-binding protein
CID	CBP-interacting domain
GST	Glutathione S-transferase
KD	Kinase-dead
